# A202 PREOPERATIVE DIAGNOSIS OF JEJUNAL ADENOCARCINOMA ASSOCIATED WITH CROHN’S DISEASE DIAGNOSED, LOCALIZED AND SURVEILLED VIA DOUBLE BALLOON ENDOSCOPY: A CASE REPORT

**DOI:** 10.1093/jcag/gwae059.202

**Published:** 2025-02-10

**Authors:** C S Liu, K Wong, K Kroeker, M Gozdzik, F Hoentjen, F Peerani, S Wasilenko, S Zepeda-Gomez, B Halloran

**Affiliations:** University of Alberta Faculty of Medicine & Dentistry, Edmonton, AB, Canada; University of Alberta Faculty of Medicine & Dentistry, Edmonton, AB, Canada; University of Alberta Faculty of Medicine & Dentistry, Edmonton, AB, Canada; University of Alberta Faculty of Medicine & Dentistry, Edmonton, AB, Canada; University of Alberta Faculty of Medicine & Dentistry, Edmonton, AB, Canada; University of Alberta Faculty of Medicine & Dentistry, Edmonton, AB, Canada; University of Alberta Faculty of Medicine & Dentistry, Edmonton, AB, Canada; University of Alberta Faculty of Medicine & Dentistry, Edmonton, AB, Canada; University of Alberta Faculty of Medicine & Dentistry, Edmonton, AB, Canada

## Abstract

**Background:**

Small bowel carcinomas (SBC) are rare, accounting for only 1% to 5% of all gastrointestinal cancers. However, patients with Crohn’s disease face a significantly elevated risk of SBC. This case report outlines a rare case of preoperatively-diagnosed jejunal adenocarcinoma in a patient with long-standing Crohn’s disease with diagnosis and surveillance through double balloon endoscopy (DBE).

**Aims:**

A 41-year-old female with a 30-year history of small bowel Crohn’s disease presents to the hospital with overt obscure GI bleeding. Throughout the course of her disease, she was thought to be in remission with Pentasa and 6-MP, though she had mild inflammation seen in the jejunal and neoterminal ileum visualized on magnetic resonance enterography (MRE). Subsequent oral DBE showed severe nodularity and friability throughout the jejunum with biopsies showing invasive moderately differentiated adenocarcinoma. Combined oral and rectal DBE was used to facilitate tattoo placement prior to open right hemicolectomy with jejunal resections, which was complicated by intraoperative bleed and anastomotic leak. Unusually, cross-sectional imaging with computer tomography (CT) and positron emission tomography (PET-CT) showed no GI tract enhancement or evidence of metastatic spread. Post-operatively, oral DBE found a saddle shaped deformity in the jejunum which had dysplasia on one biopsy. As a result of her complicated post-operative course, the patient is receiving regular surveillance with oral DBE every six months.

**Methods:**

Balloon-assisted endoscopy (BAE) has the potential to allow for endoscopic and histopathologic examination of the entire gastrointestinal tract, which makes BAE an ideal modality for both diagnosis and surveillance in Crohn’s disease. In this particular case, DBE was able to detect a subtle jejunal lesion that was not identified on CT enterography or even a PET-CT scan, resulting in earlier detection of a small bowel adenocarcinoma that was curable with surgery.

**Results:**

Currently, there is a gap in literature surrounding small bowel adenocarcinoma surveillance. This is further complicated by the lack of an established macroscopic appearance of SBC and the unknown prognostic significance of dysplasia on pathology in the context of small bowel inflammation. Further, many centres have limited access to BAE.

**Conclusions:**

This case shows that it has an important, and underutilized role, in IBD management. It is currently the only methodology for visual assessment and biopsy of lesions in the mid gut. Small bowel endoscopy access should be increased in Canada with a focus on IBD patients. Development of high risk identifiers for possible small bowel carcinoma also need to be developed as part of ongoing guidelines.

**Funding Agencies:**

None

